# Physicochemical and Sensorial Characterization of Artisanal Pasta from the Occitanie Region in France

**DOI:** 10.3390/foods11203208

**Published:** 2022-10-14

**Authors:** Loubnah Belahcen, Denis Cassan, Elodie Canaguier, Marie-Hélène Robin, Yuna Chiffoleau, Marie-Françoise Samson, Gwénaëlle Jard

**Affiliations:** 1 Food Science and Nutrition Department, INP EI-Purpan, Univ. Toulouse, CEDEX 3, 31076 Toulouse, France; 2 IATE, Univ. Montpellier, INRAE, Institut Agro, CEDEX 2, 34060 Montpellier, France; 3 AGIR, INRAE, 31326 Castanet Tolosan, France; 4 Plant Science and Agronomy Department, INP EI-Purpan, Univ. Toulouse, CEDEX 3, 31076 Toulouse, France; 5 INNOVATION, Univ. Montpellier, CIRAD, INRAE, Institut Agro, CEDEX 2, 34060 Montpellier, France

**Keywords:** artisanal pasta, cooking quality, Pivot profile, sensory analysis

## Abstract

Artisanal pasta made from wheat or underutilized cereal flours has grown in popularity with the expansion of the local and short food chains. Artisanal pasta makers do not use the same raw materials or production processes, leading to great variability in the final product. The purpose of the study is to determine the physicochemical and sensory characteristics of artisanal pasta made from durum wheat flour. Seven brands of fusilli pasta manufactured in the Occitanie region (France) were selected and analyzed in terms of their physicochemical composition (protein and ash content in dry samples), cooking properties (optimal cooking time, water absorption, and cooking loss), sensory characteristics (Pivot profile), and consumer appreciation. Differences in the physicochemical characteristics of the dry pasta samples partly explain the variations in pasta characteristics measured after cooking. The Pivot profile varied among pasta brands, but no major differences in hedonic properties were identified. To our knowledge, this is the first time that artisanal pasta made from flour has been characterized in terms of its physicochemical and sensory properties, which highlights the diversity of products on the market.

## 1. Introduction

According to French law [1], pasta products must be made exclusively from durum wheat semolina, with precise criteria for particle size, color, and ash and protein contents [2]. Since the end of the 1990s, in response to consumer demand for products perceived as healthier and more respectful of the environment, local and short production chains have been developed [3,4]. This context has favored the growth of the so-called “artisanal” pasta in food markets, with a corresponding expansion of the literature on these products since 2010 [5]. Artisanal pasta is made by small-scale processors collaborating with farmers (in cooperatives or associations) from locally produced cereals, or by farmers–processors processing their own cereals, both selling directly and locally the products. In France, more than 250,000 tons of pasta are produced each year [6], including an unspecified portion of artisanal pasta. Artisanal pasta differs from the so-called “industrial” pasta in terms of the raw materials, the milling and pasta-making processes used, leading to different nutritional, culinary and sensory qualities [7].

The types and diversity of wheats used affect the quality of the pasta produced. In the Occitanie region, artisanal pasta is produced from different durum wheat varieties, from modern breeding programs (when these are well adapted to the area or to organic farming), participatory plant breeding projects (e.g., LA1823 [8]) or from ancient wheat varieties (e.g., Bidi17). Some farmers grow other underutilized wheat species, such as einkorn, emmer, spelt, or rivet wheat. Because of the low production volume, but mostly to control all the steps in the production chain, many farmers prefer to grind their grains using stone mills a more traditional approach considered to yield healthier products.

Artisanal pasta is produced from semi- or wholemeal wheat flour instead of semolina because it is easier for small-scale processors and farmers to process it locally. Milling and especially particle size are known to have a considerable effect on pasta cooking quality [9,10] and color [11,12]. The characteristics of the end-product depend also on the drying process used. Artisanal pasta is commonly dried at low temperature (<50 °C). High temperature drying cycles (>65 °C) improve the cooking quality of pasta made from semolina [13,14] but not of pasta made from wholemeal flour. Indeed, Manthey and Schorno (2002) [15] found that wholewheat spaghetti dried at a low temperature had a better appearance and firmness than the samples dried at a high temperature despite lower cooking loss. West et al. (2013) [16] found, for wholemeal macaroni, that a short high-temperature drying process did not improve cooking quality, particularly in terms of the cooking losses measured. Unsurprisingly, the absence of standard manufacturing practices for artisanal pasta leads to a high variability in product quality.

The sensory variability of artisanal pasta can be characterized by descriptive sensory analysis (DA). The ISO 7304-1 (2016) and ISO 7304-2 (2008) [17,18] standards define three criteria (firmness, stickiness, and starch release) with which to assess the texture of pasta. A sensory attribute lexicon for dried long pasta was also recently created [19], consisting of 35 terms. However, this standardized method is time-consuming and may be unsuitable to describe artisanal short pasta made from durum wheat flour.

The so-called “alternative” sensory analysis methods, such as the Pivot profile [20], are therefore interesting as a first approach to products that have never been previously characterized by sensory analysis [21]. 

The objective of the present study is, therefore, to identify the physicochemical and sensory characteristics of artisanal pasta processed and sold in local food systems. To our knowledge, the overall quality of artisanal pasta has never been previously evaluated, particularly in terms of sensory appreciation.

## 2. Material and Methods

### 2.1. Raw Materials

**Figure 1:**
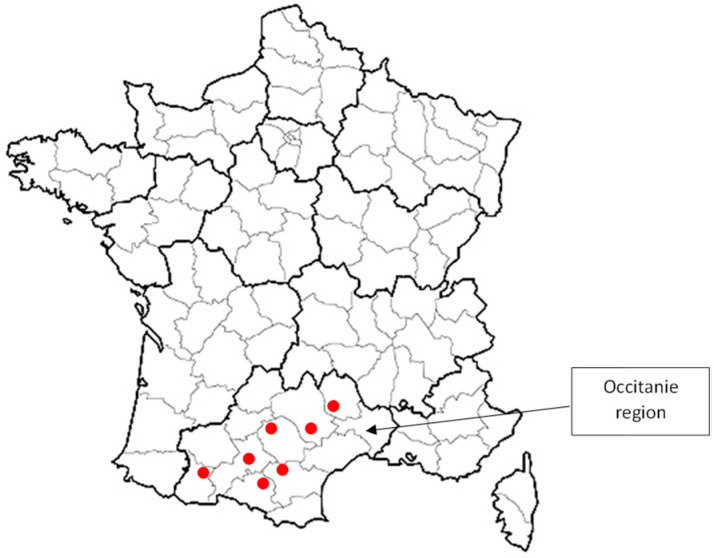
Geographical distribution of the pasta samples in Occitanie region.

Seven samples of fusilli-shaped pasta made from durum wheat flour were collected from a geographically representative distribution of pasta producers in the Occitanie region (southern France, Figure 1). These producers were chosen among the 30 registered pasta producers in the region to be representative of local artisanal pasta production, i.e., small scale (between 3 and 40 t/year), local production systems, pasta made from semi- or wholemeal flour and dried at a low temperature (between 40 and 50 °C, for 11 to 19 h).

Some of their characteristics are listed in Table 1. 

### 2.2. Physicochemical Characterization of Dry Pasta

The following properties were measured on dry samples. The **dry matter** content of the pasta was measured in triplicate according to the approved ISO 712:2009 method [23].

**Total mineral content** was determined in triplicate according to the approved ISO 2171:2010 method [24]. 

The L*, a*, b **color** coordinates of the pasta were determined using a CR410 chroma meter (Konica Minolta, Roissy, France). The pasta samples were ground (Perten Lab mill 3303, Perkin Elmer, Haguenau, France) and the powder obtained was placed in a homogenous layer inside the black box of the chroma meter. The L* component quantifies brightness from dark (L* = 0) to bright (L* = 100), a* redness, from red (+a*) to green (−a*), and b* yellowness, from yellow (+b*) to blue (−b*). Color measurements were performed in triplicate. A yellowness index (YI) was also calculated using the Francis and Clydesdales formula [25]: YI = 142.86 b*/L*. The **dimensions of the pasta** (length, width, and thickness of the pasta spirals) were measured with a caliper for 5 pasta samples. All samples were observed with an AZ100M multizoom microscope (Nikon Europe, Amsterdam, The Netherlands) with white LED epi-illumination at a low magnification (×2). 

### 2.3. Physciochemical Characterization of Cooked Pasta

#### 2.3.1. Cooking Behavior of the Pasta

**Optimal cooking times (OCTs)** were determined in triplicate according to the approved AACC Method 66-50.01 [26]. Briefly, the pasta was cooked in boiling, demineralized and salted water (7 g·L^−1^) and the OCT was defined as the time taken for the white line in the core to disappear when the pasta was crushed between two plexiglass plates, indicating that the starch had gelatinized. 

**Water absorption** was measured as the weight gain of the pasta after cooking, expressed as a percentage of the dry weight. Briefly, 100 g of pasta was cooked at OCT in 5 L of water with 7 g·L^−1^ salt. The pasta was drained, rinsed twice with tap water, and the residual water was absorbed with a paper towel before weighing. This procedure was performed twice and the average value was retained.

**Cooking losses**, i.e., dry matter losses during cooking, were calculated for each pasta sample as follows. Briefly, 8 g of dry pasta was cooked at OCT in 300 mL of water (hardness 15 ± 1 °F) in a beaker. The cooked pasta was then freeze-dried for 72 h using a Beta 2-8 LSCbasic device (Christ, Osterode am Harz, Germany) and weighed. Cooking losses were calculated as the difference in dry matter weight between the uncooked and freeze-dried cooked pasta, expressed as a percentage of the dry matter weight before cooking. 

#### 2.3.2. Texture Analyses

A TA-XTplus texture analyzer (Stable Micro Systems, Scarsdale, Godalming, United Kingdom) equipped with a TA-93WST wire mesh extrusion fixture was used to evaluate the rheological properties of the pasta after cooking. The test involved extruding a 100 g sample of cooked (OCT) and drained pasta through a wire mesh screen. The plunger height was calibrated beforehand to 110 mm above the wire mesh at the base of the extrusion cylinder. The test was conducted in compression, at 5 mm·s^−1^ and the target distance was 105 mm. The bulk of the pasta was first compacted before being extruded through the wire mesh. The average **extrusion force** was measured on the force versus time curve between 16 and 21 s, corresponding to the last 25 mm of the stroke. The test was performed twice for two samples of each brand of pasta (2 cooked samples × 2 tests per sample = 4 replicates per product).

#### 2.3.3. Protein Content and Protein Profile of Freeze-Dried Cooked Pasta

Freeze-dried cooked pasta was ground with an A10 basic mill (IKA, Staufen, Germany). The moisture content of the resulting powder was determined using AACC method 44-15.02 [26], and the **total protein** content was determined using the Kjeldahl method as described in AACC method 46-12.01 [26] with 5.7 s as the conversion factor. To determine the protein profiles, proteins were extracted following Morel et al. (2000) with modifications [27]. Freeze-dried and ground cooked pasta samples (160 mg) were suspended in 20 mL of sodium phosphate buffer (0.1 M, pH 6.9) containing 1% (*w*/*v*) sodium dodecyl sulfate (SDS). The suspension was stirred for 80 min at 60 °C. After centrifugation (39,000× *g*; 30 min; 20 °C), the supernatant containing SDS soluble proteins was collected and stored (−20 °C) until analysis. The pellet was re-suspended in 5 mL SDS–sodium phosphate buffer containing 20 mM dithioerythritol (DTE) and sonicated for 3 min at 7.5 watts. The new supernatant was stored until analysis. The proteins recovered after the different extraction steps were separated by size-exclusion high-performance liquid chromatography (SE-HPLC) using a TSKgel G4000 SWXL column (7.8 mm i.d. × 30 cm, TOSO BIOSCIENCE GmbH, Griesheim, Germany), following Dachkevitch and Autran (1989) [28] on an Alliance system (Waters, Saint Quentin en Yvelines, France). The proteins were eluted at ambient temperature with 0.1 M sodium phosphate buffer (pH 6.9) containing 0.1% (*w*/*v*) SDS at a flow rate of 0.7 mL·min^−1^ and the absorbance was measured at 214 nm. The fraction of **SDS-soluble proteins** was obtained from the area under the first peak in the chromatograms obtained, and the fraction of **DTE-soluble proteins** (i.e., after DTE reduction and sonication) from the area under the second. Both areas were converted into protein contents and the non-extracted protein fraction was calculated by subtracting the sum of the SDS-soluble and DTE-soluble protein contents from the Kjeldahl total protein content. When the fraction of non-extracted protein was negative, due to high recovery, this value was forced to 0 and the sum of the SDS soluble and DTE extracted protein fractions was corrected to reach 100%.

#### 2.3.4. In Vitro Pasta Digestibility Tests

The digestibility of cooked pasta samples was evaluated by measuring the rate of proteolysis in vitro using the Protein Digestibility Assay kit (Neogen, Auchincruive, UK), with a few modifications to the standard procedure. For each brand of pasta, proteolysis was carried out on two 250 mg samples, according to manufacturer specifications and trypsin/chymotrypsin digestion was conducted for 4 h. Digestion was stopped by immersing the tubes in boiling water. After cooling, the tubes were centrifuged at 4696× *g* for 15 min at 15 °C. After centrifugation, the supernatants were set aside and the pellets frozen. Protein digestibility was then estimated by determining the amount of nitrogen remaining in the pellets from two extractions of the same sample using the Kjeldahl method. The extent of proteolysis after 1 h of peptic digestion followed by 4 h of tryptin/chymotryptin digestion was expressed as the percentage of the initial protein content of the sample that remained after digestion.

### 2.4. Sensory Analysis

#### 2.4.1. Descriptive Sensory Analysis

Pivot profiling was carried out as described by Thuillier et al. (2015) [20]. Fifty-seven panelists aged 20 to 50 years (58% female) were recruited. Among the panelists, 28.5% consumed regularly artisanal pasta, 34.6% consumed artisanal pasta once in a while, and 36.7% never consumed artisanal pasta. At the beginning of each session, participants confirmed they were willing and consented to participate and that they did not have any food allergies.

Ten-gram samples of cooked pasta (OCT) were served to each panelist on white plates, at room temperature. Sensory analysis was carried out in separate boxes under white light. The samples were coded with three-digit random numbers and were presented in sequential monadic order by pair (one sample and one pivot). A Latin square design was used to balance the sample order. 

Before the start of the sensory analysis session, pasta brand 3 (Pasta 3) was chosen as the pivot because it was the most “central product” in terms of color, texture, and taste. Participants evaluated the samples according to three criteria (visual appearance, in-mouth texture, and flavor) with free comments (attributes) as “more” or “less” than the pivot. Negations and hedonic comments were avoided. 

All attributes were listed and categorized and then grouped by synonyms after discussions with panelists and a dictionary of synonyms was generated using the software TASTEL (version 2015.2, ABT informatique, Rouvroy-sur-Marne, France) The main descriptors were selected by frequency of citation. The number of negative comments were subtracted from the number of positive comments for each descriptor and the resulting scores were adjusted to obtain only positive scores. A contingency table was obtained, and the pivot was integrated by attributing scores of 0 before adjustment, as described by Fonseca et al. (2016) [29].

#### 2.4.2. Ranking Test

A ranking test was performed according to NF ISO 8587 to classify the pasta samples in terms of hedonic properties [30]. Sixty-five pasta consumers aged 19 to 60 years (55% female) were recruited. At the beginning of each session, participants confirmed they were willing and consented to participate and that they did not have any food allergies. Ten-gram samples of cooked pasta (OCT) were served to each panelist on white plates, at room temperature. Sensory analysis was carried out in separate boxes under white light. Samples coded with three-digit random numbers were presented simultaneously to the participants and ranked from most (rank 1) to least preferred (rank 7).

### 2.5. Statistical Analyses

All statistical analyses were performed with XLSTAT (Addinsoft, Paris, France). The results were considered statistically significant at *p* < 0.05.

The results for the **physicochemical characteristics of dry and cooked pasta** (moisture, ash, color, firmness, OCT, and water absorption) were compared using Kruskal–Wallis tests. For these analyses, multiple pairwise comparisons were performed with the Conover/Iman test. Correlations between parameters were measured using Pearson correlation matrices and principal component analysis (PCA). 

A sensory map was generated from the **Pivot profile** contingency table using correspondence analysis (CA). Correlations between descriptors and products were investigated using a global chi-squared test and, if this was significant, chi-squared tests were performed cell-by-cell as previously described by Fonseca et al. (2016) [29]. 

The **ranking test** data were analyzed using Friedman’s test.

## 3. Results

The physicochemical characteristics of the different brands of pasta (dry and cooked samples) are listed in Table 2.

### 3.1. Dry Pasta Characterization

The moisture content of the pasta was below 12.5% in all cases, in accordance with French regulations. Mineral contents were high and ranged from 1.00 to 1.88 % DM, with some samples containing more than the maximum of 1.3 % recommended in France for durum wheat semolina and pasta [1]. 

**Figure 2:**
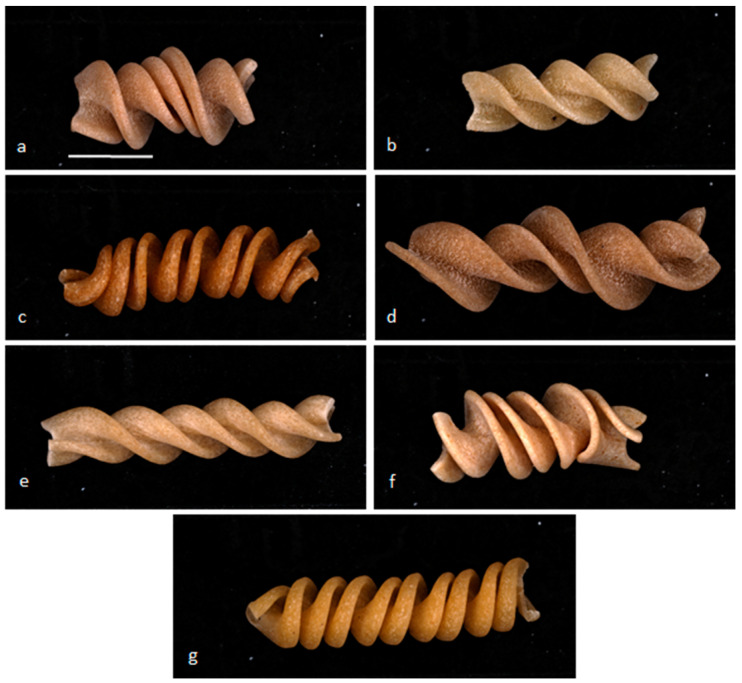
Diversity of shape and appearance of different brands of fusilli pasta: photographs of (**a**) pasta 1, (**b**) pasta 2, (**c**) pasta 3, (**d**) pasta 4, (**e**) pasta 5, (**f**) pasta 6, and (**g**) pasta 7 (white bar = 1 cm).

The shape of the fusilli varied between brands. Some were shorter than others (approximately 25 mm for pasta 1, 2, 4, and 6 versus approximately 30 mm for pasta 3, 5, and 7). Samples 2, 3, 5, and 7 were more regular in shape and narrower than samples 1, 4, and 6. The surfaces of pasta samples 1, 2, 4, and 6 were irregular and rough, a typical characteristic of pasta prepared with bronze extrusion dies. White specks were observed on pasta samples 2, 3, 5, and 7 (Figure 2), probably due to insufficient hydration [31]. Samples 2, 3, 6, and 7 also had black/brown specks (black point disease and bran particles).

In terms of L*, a*, b* characteristics (Table 2), the brightness (L*) of the pasta ranged from 66.88 to 75.78. Samples 2 and 7 were brighter, samples 3 and 4 darker, and samples 1, 5, and 6 intermediate. The redness (a*) component ranged from 2.03 to 4.76 and none of the pasta samples were similar in this respect. Yellowness (b*) ranged from 19.66 to 24.22, with samples 3 and 7 being the most yellow and samples 1 and 2 being the least yellow. The highest YI was measured in pasta 3 and the lowest in pasta 5. 

### 3.2. Cooking Behavior of the Pasta

The optimal cooking times ranged from 6 to 11.5 min. Pastas 5 and 3 had the shortest OCTs, pastas 6 and 1 the longest, and the other three brands had OCTs close to the overall mean (Table 2). 

No significant differences in texture were observed among pasta samples 1, 2, 4, and 5 and among samples 3, 6, and 7. The first group was firmer (mean firmness, 93.79 N) than the second (mean firmness, 66.12 N). Water absorption ranged from 123.9% to 196.7%, with, as for the firmness, two groups: samples 3, 6, and 7 with a mean water absorption of 194.1% and samples 1, 2, 4, and 5 with a mean water absorption of 150.2%. Pasta 4 had the lowest water absorption and was among the firmest.

Pasta cooking losses ranged from 11.20 to 15.01 % with an average value of 12.86 ± 1.56%. 

### 3.3. Protein Profile and Protein In Vitro Digestion

The protein content of the pasta ranged from 9.84 to 13.39 g/100 g DM, with a mean of 11.65 ± 1.26 g/100 g DM. Pasta 6 had a much lower protein level than that expected for durum wheat pasta. The percentages of SDS-soluble, DTE-soluble (after sonication), and unextractable proteins are reported in Table 2. All the samples of cooked pasta had high concentrations of aggregated proteins, as shown by the high percentages of DTE-soluble and unextractable proteins. Low SDS-soluble protein fractions may indicate the formation of additional disulfide bonds during processing and cooking. Pasta 4 had the highest concentration of SDS-soluble protein (39.82 %) and pasta 7 the lowest (21.43%), both indicating a high degree of protein aggregation during processing and cooking. All samples except for pasta 3 and 4 (0.03 and 2.78%, respectively) had high concentrations of unextractable proteins. In the protein digestibility assays, pasta 4 differed significantly from the other samples with a much lower level of proteins remaining after 5 h proteolysis (64.37%).

### 3.4. Correlation between Parameters

Several of the physical and chemical parameters in the Pearson matrix were significantly associated (*p* < 0.05). Cooking losses were significatively correlated with ash contents (*p* = 0.02). Firmness and water absorption were negatively correlated. The protein content in the cooked pasta was negatively correlated with YI and was positively correlated with protein content after 5 h proteolysis. Firmness was not associated with protein content. Cooking losses were negatively, but not significantly, correlated with firmness (correlation factor, −0.754; *p* = 0.05).

**Figure 3:**
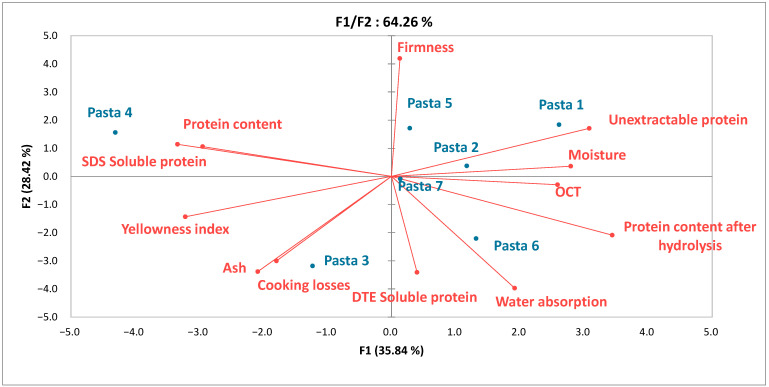
PCA plot of the pasta samples in terms of their physicochemical characteristics.

The first two principal components (Figure 3) accounted for 64.26% of the variability of the data. Pasta 4 was characterized by a high level of soluble proteins, high YI, low water absorption, and low remaining protein content after 5 h proteolysis. Pasta samples 1, 2, and 5 were distinguished by greater firmness, lower cooking losses, and lower mineral content. Samples 3 and 6 were softer, with higher water absorption, ash content, and cooking loses. Pasta 7 had intermediate values of a number of features (ash content, YI, firmness, and OCT). 

### 3.5. Sensory (Pivot Profile) Analysis

**Figure 4:**
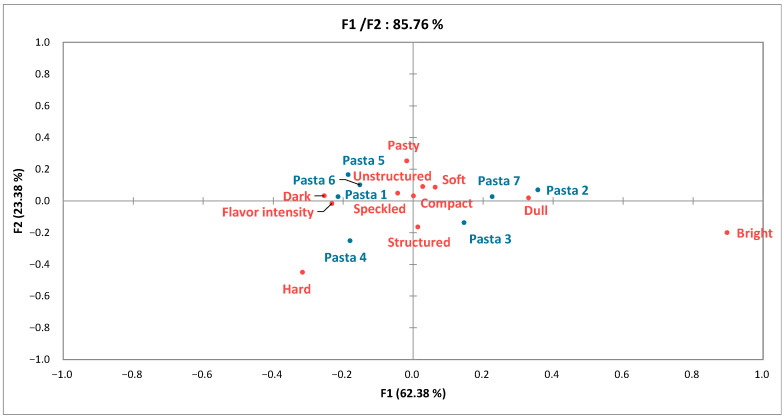
Correspondence analysis of the contingency table obtained from the Pivot analysis.

The results of the Pivot profile analysis were summarized in a contingency table (Table 3) and a CA map (Figure 4). The words used by participants to describe the samples (1485) were analyzed and grouped by meaning. Fifteen descriptors accounted for 79% of the terms used and were classified as follows: seven terms describing the appearance of the pasta (bright, yellow, dark, structured, speckled, unstructured, and compact), five terms for the texture (hard, pasty, soft, grainy, and melting), and three terms for the flavor (dull, flavor intensity, and salty).

The chi-squared test performed on the contingency table was significant (*p* < 0.001); therefore, further chi-squared tests were performed on a cell-by-cell basis to investigate associations between descriptors and specific samples [29]. Results are shown alongside the data in Table 3. In terms of appearance, samples 2 and 7 were considered brighter and samples 1, 5, and 6 less bright. Pasta 4 was deemed more yellow and structured. There were no significant differences between samples in terms of the descriptors speckled, unstructured, and compact, which were therefore not discriminating for these products. In terms of texture, pastas 1 and 4 were classified as harder and pastas 2 and 7 less hard. Pasta 4 was judged to be less pasty and pasta 5 more pasty than the others. The descriptors grainy, soft, and melting were not differentiating for these samples, despite having been used frequently by participants. In terms of flavor, samples 2 and 7 were considered duller, whereas pastas 4 and 5 were described as being more intense in flavor. Finally, “salty” was not a differentiating descriptor in this analysis. 

The first two dimensions of the CA accounted for 85.76% of the variability of the data. Pasta 3 (the pivot) was integrated into the contingency table for the map representation (Figure 4). Pasta 4 stood out from the other through its hard, non-pasty texture and more intense flavor. Samples 1, 5, and 6 formed a cluster and of more colorful and intensely flavored pasta. Samples 2 and 7 were less dark and were duller in flavor. 

### 3.6. Sensory Appreciation

**Figure 5:**
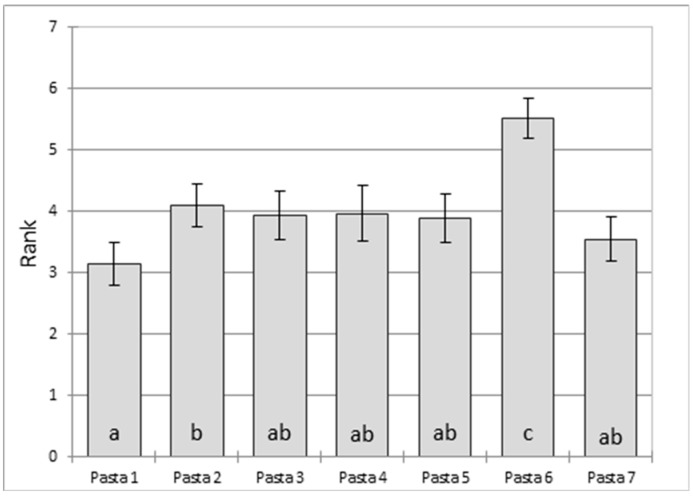
Results of ranking tests based on hedonic characteristics. a–c Mean values labeled with the same letter are not significantly different (*p* > 0.05).

Pasta 6 was the least appreciated pasta and pasta 1 the most appreciated (Friedman test, Figure 5). There were no significant differences in rank between pasta samples 3, 4, 5, and 7 and pasta 1, or between this group and pasta 2.

## 4. Discussion

The aim of this study was to characterize artisanal pasta made from durum wheat flour produced in the Occitanie region in France. Samples of dry and cooked pasta were analyzed. The literature on industrially produced pasta quality is vast, especially for durum wheat spaghetti made from semolina [5,32,33]. To our knowledge, however, no study has previously been performed on the physicochemical properties and sensory quality of artisanal pasta made from durum wheat flour.

The cooking and the organoleptic qualities of pasta are known to depend on the physicochemical characteristics of the durum wheat flour or semolina used (ash, protein, and color) and on the specifics of the manufacturing process (milling, hydration, mixing, extrusion, and drying) [33]. The pasta-making process should lead to the formation of a protein network (gluten) that entraps starch granules and prevents their leaching during cooking to produce pasta with a compact structure [34]. The characteristics of cooked pasta (firmness, dry matter loss, and water absorption) can be explained in part by its physicochemical properties before cooking (especially its ash and protein content), whose variability explains the diversity of products encountered. The pasta samples studied here were produced from semi-whole or wholemeal flour, as reflected by their rather high ash contents (mean, 1.47%; range, from 1.00 to 1.88%). Industrially produced pasta is generally made from durum wheat semolina with low ash contents (0.6–0.9%) [35]. In accordance with the results from Pearson correlation matrix, the high ash content led to high cooking losses, high water absorption, and low firmness, probably because of bran particles weakening the protein network [5]. The protein content measured in our study was in average of 11.65 % DM, in the range of the industrial pasta protein content encountered in the literature [19,35]. A high protein content is required to produce pasta with good cooking quality [33]. The quantity but also the quality of proteins affects textural properties [22]. Indeed, pasta with a low protein content and less gluten will not have a protein network capable of preventing the leaching of starch granules during cooking and will therefore be stickier. A high protein content is a key parameter in the drying process even more impactful at low temperatures. D’Egidio et al. (1990) found indeed that protein content and gluten quality both played a crucial role if a low drying temperature was used, whereas with high-temperature drying, gluten quality was less important than protein content [36]. No direct correlation between pasta firmness and both quantity and quality of protein was observed in our study. Moreover, data obtained on the degree of gluten polymerization and its susceptibility to proteolysis after cooking suggest that pasta samples differ in the structure of their gluten networks as previously explained by Petitot et al. [27] and Bruneel et al. [37] with less accessible gluten networks corresponding to firmer pasta. It could be explained by different processing parameters (mixing, extrusion, and drying) that are well known to affect the gluten network in pasta and thus its firmness [5].

Color is an important purchasing criterion for pasta consumers and a discriminating factor between brands [38]. The studied samples varied in color from bright yellow to dull brown. No direct correlation was found between the brightness, redness, and yellowness (L*, a*, b*) of the dry samples and the physicochemical variables considered. The color of pasta depends on several biochemical and technological factors, such as the quantity of yellow pigments and soluble brown pigments, the activity of enzymes such as polyphenol oxidase and peroxidase, the protein content, the ash content, and the particle size of the flour [39,40,41]. For example, pasta made from flour tends to be brighter and less yellow [12], but high ash and protein contents have a negative effect on brightness [42]. The observed variety of colors can therefore be explained by different combinations of these parameters. 

Descriptive analyses have widely been used to study the relationship between pasta production parameters and sensory quality. The effects of new cereals (e.g., emmer) have been studied by Kucek et al. (2017), for example [43]. The impact of process parameters such as drying has been investigated by Padalino et al. (2016) and West et al. (2013) [14,16]. Many studies have also been performed on the use of alternative ingredients and their effect on nutritional and sensory characteristics [44]. 

The differences in the appearance of the pasta samples and their variable cooking qualities were highlighted by Pivot profile. This alternative sensory analysis method has previously been validated for the comparison of a set of products with respect to each other. The reliability of our results is supported by the well-balanced nature of the panel (58.5% female, 41.5% male) with roughly a third each of regular, occasional, and not consumers of artisanal pasta (28.5, 34.6, and 36.7%, respectively). The panel was also sufficiently large (n = 57). Ares (2015) have shown indeed that at least 50 untrained panelists are required in this context to obtain reproducible results [45].

The Pivot profile method, based on free consumer descriptions, is simple for participants, fast, and robust [20], and has been validated for the sensory description of a set of products with respect to each other, in comparison with other alternative sensory analysis methods [29,46,47,48]. For example, Esmerino et al. (2017) [46] compared Greek yogurt samples using the Pivot profile, the projective mapping (PM), and the check-all-that-apply (CATA) questions and found that the Pivot profile was closer to similarity-based methods, such as projective mapping, than to verbal-based approaches, such as check-all-that-apply, suggesting that it is well-suited for general product descriptions. This alternative method was an appropriate choice for our study because (i) it allowed consumers to generate descriptors for previously undescribed products, and (ii) it revealed the main overall differences between the studied products. It does not provide detailed sensorial description of each pasta, on the contrary to a descriptive analysis using, for example, the lexicon from Irie et al. [19]. This lexicon consists of 35 attributes: 5 for visual appearance, 11 for aroma flavor, and 19 for texture. In comparison, the ratio of attributes in the Pivot profile suggests our panelists were more comfortable describing visual appearance (7/15 attributes) and texture in mouth (5/15 attributes). The proportion of flavor attributes (3/15 attributes) is similar to the one in Irie et al.’s lexicon (11/35 attributes). Our panelists chose less precise qualifiers (dull, flavor intensity, and salty) than found in Irie et al.’s lexicon (wheat aroma, wheat flavor, sweet aroma, roasted aroma, deteriorated grain, cinnamon, bran, pungent, corn, astringent, and chlorine), leading to a less precise sensory characterization. Significant differences between pasta types were nevertheless identified for more than half of these attributes (8/15 attributes). A previous study of spaghetti involving a trained panel found, as did we, that the main discriminating attributes were related to texture (firmness, elasticity, and stickiness) [35]. No correlation was observed between firmness and sensory attributes. This is probably because texture was defined by several attributes (pasty, hard, and compact) in the Pivot profile. 

Since all the pasta samples have been mapped at the sensory level, it would be interesting to use the attributes generated by the Pivot method to analyze each type of pasta using the same descriptive approach as Khalil et al. (2022) for a variety of labneh, a typical Lebanon fermented milk [49]. This could provide elements to better understand the hedonic evaluation.

## 5. Conclusions

In the context of an evolving market and the growing popularity of locally sourced products, this study provides information on the physicochemical and sensory characteristics of artisanal pasta processed from durum wheat flour and sold locally by producers, a previously unstudied topic. The studied pasta samples were all sourced from a specific geographical area, but our results highlighting the variable physicochemical and sensory nature of artisanal pasta are generalizable to other territories. The variability observed was slightly correlated to physico-chemical characteristics but finally not perceived by the consumers. The cooking quality of artisanal pasta on some aspects (e.g., cooking losses) should be improved. Helping pasta producers optimize the cooking quality of these kinds of pasta will require identifying more specific relationships between input variables and product properties at the artisanal level.

## Figures and Tables

**Table 1:** Characteristics of the studied pasta samples.

Sample Number	Unit of Production	Type of Agriculture	Type of Wheat Used	Type of Milling	Die Material
1	Small-scale cooperative processing unit	Organic	Rivet wheat *^,o^	Stone milling	Bronze
2	Individual small-scale farmer–processor	Organic	Bidi17 ^o^, Senatore Capelli ^o^, LA1823 ^m^	Stone milling	Bronze
3	Association of farmers and small-scale miller and pasta maker	Conventional	Voilur ^m^, Anvergur ^m^	Stone milling	Teflon
4	Association of farmers and small-scale miller and pasta maker	Organic	Atoudur ^m^	Stone milling	Bronze
5	Individual small-scale farmer–processor	Organic	LA1823 ^m^, Anvergur ^m^	Roller milling	Teflon
6	Association of farmers and small-scale miller and pasta maker	Organic	Own mix of durum wheat (~40 varieties) mixed at sowing time	Stone milling	Bronze
7	Association of farmers and small-scale pasta maker	Conventional	Miradoux ^m^, Anvergur ^m^	Roller milling	Teflon

^o^: old durum wheat; ^m^: modern durum wheat as defined by Mefleh et al., 2019 [22]. *: Rivet wheat (*Triticum turgidum* L. ssp. turgidum) is considered a specific type of half-vitreous durum wheat different from other forms of durum wheat (*Triticum turgidum* L. ssp. durum).

**Table 2:** Main characteristics of the dry and cooked pasta.

Samples	Dry Pasta						Cooked Pasta								
	Moisture (%)	Ash (g/100 g DM)	Color				Firmness (N)	OCT (min)	Water Absorption (%)	Cooking Losses (%)	Protein Content (g/100 g DM)	SDS-Soluble Protein (%)	DTE-Soluble Protein (%)	Unextractable Protein (%)	Protein Content After Proteolysis (%)
			L*	a*	b*	Yellowness Index									
Pasta 1	8.79 ^a^	1.00 ^a^	70.09 ^bc^	3.95 ^d^	19.93 ^a^	40.62 ^bc^	89.80 ^a^	11.50 ^f^	158.3 ^abc^	11.20	11.25	24.59	60.41	14.99	79.34
Pasta 2	8.30 ^ab^	1.56 ^abc^	74.46 ^de^	2.84 ^b^	19.66 ^a^	37.73 ^a^	99.75 ^a^	8.85 ^cd^	166.1 ^bc^	11.47	11.32	22.62	66.80	10.58	80.29
Pasta 3	8.12 ^bc^	1.63 ^bc^	67.46 ^ab^	4.76 ^g^	23.26 ^c^	49.25 ^e^	61.06 ^c^	7.16 ^a^	196.3 ^d^	14.25	11.92	28.02	71.96	0.03	80.82
Pasta 4	7.38 ^d^	1.66 ^bc^	66.88 ^a^	4.24 ^e^	22.15 ^bc^	47.31 ^cd^	91.05 ^a^	7.53 ^bc^	123.9 ^a^	14.02	13.39	39.82	57.40	2.78	64.37
Pasta 5	8.11 ^bc^	1.30 ^ab^	67.36 ^a^	4.44 ^f^	22.62 ^bc^	47.96 ^de^	94.57 ^a^	6.00 ^a^	152.7 ^ab^	11.50	10.76	31.71	58.30	9.99	79.82
Pasta 6	7.99 ^cd^	1.88 ^c^	74.01 ^cd^	2.95 ^c^	20.62 ^ab^	39.80 ^ab^	63.88 ^bc^	11.00 ^ef^	196.7 ^d^	15.01	9.84	27.62	60.98	11.40	81.84
Pasta 7	7.18 ^d^	1.29 ^ab^	75.78 ^e^	2.03 ^a^	24.22 ^c^	43.77 ^bc^	73.43 ^b^	9.83 ^de^	189.4 ^cd^	12.56	13.07	21.43	61.63	16.93	75.35
Mean	7.98	1.47	70.86	3.60	21.78	43.96	81.93	8.84	169.0	12.86	11.65	27.97	62.50	9.53	77.40
SD	0.55	0.29	3.82	1.00	1.74	3.56	15.58	2.05	26.9	1.56	1.26	6.29	5.15	6.13	6.10

^a–f^ Mean values labeled with the same letter(s) in the same column are not significantly different (*p* > 0.05) according to Conover/Iman tests.

**Table 3:** Contingency table obtained from the Pivot analysis.

Category	Descriptors	Pasta 1	Pasta 2	Pasta 4	Pasta 5	Pasta 6	Pasta 7
Visual Appearance	Bright	6 (−)	52 (+)	11	0 (−)	2 (−)	46 (+)
	Yellow	43	61	61 (+)	46	52	37
	Dark	65 (+)	19 (−)	47	60	61	42
	Structured	38	43	63 (+)	40	38	48
	Speckled	61	50	51	57	61	58
	Unstructured	54	55	37	53	51	48
	Compact	51	47	42	48	50	48
Texture in mouth	Hard	43 (+)	5 (−)	53 (+)	19	27	12 (−)
	Pasty	55	46	13 (−)	60 (+)	51	35
	Soft	52	60	42	55	55	60
	Grainy	61	52	55	53	57	48
	Melting	42	57	51	54	53	56
Flavor	Dull	21 (−)	53 (+)	22 (−)	25	35	45 (+)
	Flavor intensity	70	35 (−)	74 (+)	76 (+)	64	49
	Salty	48	52	49	56	53	51

(−/orange color) and (+/green color) indicate significant results (*p* < 0.05, chi-squared test).

## Data Availability

The data presented in this study are available on request from the corresponding author. The data are not publicly available due to adherence to the General Data Protection Regulation of the European Union.
